# Comparison of the effects of esketamine/midazolam and remifentanil/midazolam on respiratory mechanics in mechanically ventilated patients with acute respiratory distress syndrome

**DOI:** 10.1186/s12871-025-03211-x

**Published:** 2025-07-05

**Authors:** Dujuan Qiao, Wei Liu, Huanjia Xue, Rui Liu, Ya Gao, Jingzhi Dong, Wei Wu, Junkai Feng, Min Li, Linong Yao

**Affiliations:** 1 Department of Critical Care Medicine, Tangdu Hospital, Air Force Medical University, Xi’an, Shaanxi, China; 2 Department of Anesthesiology & Perioperative Medicine (D), Xi’an People’s Hospital (Xi’an Fourth Hospital), Xi’an, Shaanxi China; 3 Department of Anesthesiology, Tangdu Hospital, Air Force Military Medical University, Xi’an, Shaanxi China; 4 Department of Anesthesiology, Xi’an Hospital of Traditional Chinese Medicine, Xi’an, Shaanxi, China

**Keywords:** ARDS, Esketamine, Respiratory mechanics, Mechanical ventilation

## Abstract

**Background:**

Esketamine exerts analgesic effects and has pharmacological properties of bronchodilation and elevation of mean arterial pressure, making it an interesting analgesic agent for patients with ARDS. Therefore, we conducted a randomized controlled trial comparing the effects of esketamine/midazolam and remifentanil/midazolam on respiratory mechanics and oxygenation in mechanically ventilated patients with ARDS.

**Methods:**

In this prospective, randomized, controlled study, patients with ARDS who were undergoing mechanical ventilation were randomly assigned to receive either remifentanil/midazolam (*n* = 25) or esketamine/midazolam (*n* = 25). Both groups maintained equivalent levels of sedation and analgesia. The primary outcomes were airway resistance (R_aw_) and static respiratory system compliance (C_st_) at 24, 48, and 72 h post-medication administration. Plateau Pressure (P_plat_), peak airway pressure (P_peak_), hemodynamic parameters, mechanical ventilation duration, and ICU length of stay were also recorded.

**Results:**

C_st_ at 72 h post-medication in esketamine group (49.8 ± 13.8) was higher than that in control group (42.4 ± 11.9) (*P* < 0.05). There was no significant difference in R_aw_ at 24 h, 48 h and 72 h post-medication between the two groups (*P* > 0.05). At 72 h post-medication, both P_plat_ and P_peak_ in the esketamine group [(15.6 ± 3.1); (25.7 ± 3.8)] were significantly lower than those in the control group[(17.7 ± 4.2); (28.5 ± 4.7)] (*P* < 0.05). Additionally, the esketamine group [(256.6 ± 89.1); (266.4 ± 88.4); (284.92 ± 84.45)] demonstrated significantly higher PaO₂/FiO₂ ratios compared to the control group [(208.7 ± 61.5);(219.52 ± 61.28); (222.00 ± 68.54)] at all measured time points (*P* < 0.05). MAP and heart rate were higher in the esketamine group than in the control group, while cumulative doses of vasoactive drugs were comparatively lower in the esketamine group. The duration of mechanical ventilation and ICU length of stay did not-differ (*P* > 0.05).

**Conclusion:**

Esketamine/midazolam leads to improved in C_st_ and PaO_2_/FiO_2_ ratio compared to remifentanil/midazolam in mechanically ventilated patients with ARDS.

**Clinical trial registration:**

This study was registered at Chinese Clinical Trial Registry (ChiCTR2300070733) on April 21, 2023. URL: https://www.chictr.org.cn/.

**Supplementary Information:**

The online version contains supplementary material available at 10.1186/s12871-025-03211-x.

## Introduction

Acute respiratory distress syndrome (ARDS) is prevalent in 10.4% of critically ill patients admitted to the intensive care unit (ICU), and in more than 20% of those on invasive mechanical ventilation, with the mortality rate reaching as high as 35–46% [1]. Furthermore, the high morbidity and mortality of this severe lung condition present prominent treatment challenges.

Currently, no specific treatment exists for ARDS, with invasive mechanical ventilation being the primary approach. Moreover, the lungs of patients with ARDS are mechanically heterogeneous, while atrophic atelectasis in the gravity-dependent and hyperinflation in the non-gravity-dependent regions of the lungs render these patients more susceptible to mechanical ventilation-related lung injury. In previous studies, clinicians have focused more on oxygenation changes in patients with ARDS and neglected modulation in lung stress. Lung injury is invariably accompanied by alterations in the mechanical properties of the lungs. Correspondingly, altered lung mechanics can be highly indicative and specific to the nature and degree of lung injury [2] making respiratory mechanics an extremely valuable bedside tool. Several respiratory parameters can be obtained at the bedside to provide measurements of lung stress, strain, and heterogeneity, thereby guiding mechanical ventilation and improving respiratory conditions [3]. Except the adjustment of ventilator parameters, adjuvant therapy during mechanical ventilation in patients with ARDS may also reduce the impact of mechanical ventilation.

Analgesia and sedation management is one of the core treatments during mechanical ventilation. A rational analgesia and sedation strategy is patients with ARDS can lower oxygen consumption, promote patient-ventilator synchrony, improve oxygenation, and prevent or mitigate ventilator-associated lung injury [4]. Currently, opioids are the primary drugs utilized in ICU analgesic management. Among them, remifentanil is a short-acting drug that offers a fast onset of action, strong analgesic effect, rapid recovery after drug withdrawal, and good controllability. However, this opioid drug is associated with a frequent incidence of adverse effects [5]. In particular, opioids inhibit the respiratory center, thereby causing patients to become unable to maintain spontaneous breathing during mechanical ventilation and making it difficult to clear deep-lying phlegm [6]; these drugs also activate µ-receptors to stimulate motor fibers in the vagus nerve, cause bronchoconstriction, and heighten airway resistance(R_aw_), ultimately leading to alveolar collapse and atelectasis, reduced static respiratory system compliance (C_st_), and diminished oxygenation [7]; additionally, opioids impair immune responses, thus increasing the likelihood of pulmonary infections in these patients [8]; lastly, these drugs enhance the function of the gastrointestinal vagus nerve to reduce smooth muscle contraction, elevate sphincter contraction activity, and slow gastrointestinal motility [9], resulting in increased abdominal pressure and restricted chest wall compliance and reduced lung compliance. All these opioid use-associated alterations in respiratory parameters can aggravate the ARDS condition in these patients.

Several recent guidelines have recommended ketamine as an analgesic agent in critically ill patients in the ICU [10, 11]. Ketamine is an anesthetic drug that induces analgesia via the non-competitive inhibition of NMDA receptors. R-ketamine and S-ketamine are the optical enantiomers of ketamine. Esketamine, which is essentially S-ketamine, possesses pharmacological characteristics similar to those of ketamine, but with higher potency. As described earlier, esketamine exerts its analgesic effects by inhibiting NMDA receptors; leading to the impairment of the spinoreticular pathway at the spinal cord level and inhibition of the large‐conductance Ca^2+^‐activated K^+^ channels [12]. During the exertion of its analgesic effects, esketamine simultaneously blocks the L-type Ca^2+^ channels to relax bronchiolar muscle activity and produces sympathomimetic effects by inhibiting neuronal norepinephrine reuptake [13]. All these pharmacological effects of esketamine theoretically lower R_aw_ and enhance respiratory drive, which results in alleviated atelectasis, increased C_st_, and improved respiratory mechanics. Therefore, ketamine usage may be advantageous to critically ill patients undergoing mechanical ventilation. Prior studies have also demonstrated that continuous ketamine infusion in pediatric patients with refractory bronchospasm undergoing mechanical ventilation significantly improves C_st_ and oxygenation [14]. Similarly, ketamine administration in mechanically ventilated patients from surgical ICUs has been found to increase inspiratory flow and diminish respiratory effort [15]. However, limited evidence is available on the application of esketamine to improve respiratory mechanics in the ICU setting, particularly in patients with ARDS.

The short-acting opioid drug remifentanil combined with midazolam is commonly employed for analgesia and sedation in ICU patients. Prior studies have shown that the combined administration of ketamine and benzodiazepines, such as midazolam, produces analgesic, sedative, and amnesic effects, as well as reduces neurological and cardiovascular side effects. We conducted a randomized controlled trial to identify an analgesic and sedative strategy with less respiratory impact on critically ill patients undergoing mechanical ventilation in the ICU and to optimize the management strategy of mechanical ventilation. We hypothesized that esketamine would have a lower respiratory impact on mechanically ventilated patients with ARDS than remifentanil, which would be reflected by improvements in respiratory mechanics parameters and the subsequent enhancement of oxygenation.

## Materials and methods

The manuscript was written in accordance with the CONSORT statement guideline for a randomized controlled trial.

### Study protocol and approval

This study protocol was approved by the Ethics Committee of Tangdu Hospital of Air Force Military Medical University (approval NO. K202302-19; title: “Effect of analgesic sedation with esketamine combined with midazolam on respiratory mechanics in mechanically ventilated patients”; approval date: March 3, 2023). This study was registered at Chinese Clinical Trial Registry (ChiCTR2300070733) on April 21, 2023. The procedures used in this study were in accordance with the principles of the Helsinki Declaration. The study protocol was explained to the patients’ legal representative before trial initiation and written informed consent was obtained from them.

### Inclusion and exclusion criteria

Patients with a diagnosis of ARDS according to the diagnostic criteria of the 2012 Berlin definition and undergoing mechanical ventilation [16] (ARDS patients undergoing endotracheal intubation and mechanical ventilation within 24 h of ICU admission and expected to last longer than 72 h) with a requirement for sedation and analgesia management were included. Patients were excluded if they met any of the following criteria: age < 18 years; documented allergy to esmolol, midazolam, or remifentanil; a history of dementia or psychiatric illness; use of antipsychotic or antidepressant medication at home; lung lobectomy; rib fractures requiring pressure fixation; poorly controlled blood pressure (grade 3 hypertension) or tachycardia (heart rate > 150 beats/min); suspected or confirmed intracranial hypertension; or undergoing extracorporeal membrane oxygenation or prone positioning ventilation therapy.

### Randomization and blinding

This study was a prospective, randomized, controlled, double-blind trial conducted at the ICU of Tangdu Hospital, Air Force Medical University. One researcher generated 50 random numbers using a computer, and the patients were divided into a control or esketamine group in a 1:1 ratio. These random numbers were then placed into 50 envelopes. After a patient was enrolled, a medication nurse randomly selected an envelope and prepared the experimental drug concentration for the patient according to the group allocated to the random number. Further, a bedside nurse covered the infusion pump and adjusted the infusion rate based on the individual Richmond Agitation-Sedation Scale (RASS) and Critical-Care Pain Observation Tool (CPOT) scores. Only the medication and bedside nurses were aware of the group assignment of the patients, whereas the remaining researchers such as clinicians, statisticians and patients were blinded to the group allocation.

### Treatment protocols

All enrolled patients underwent cardiac monitoring, radial artery puncture, the lung injury score (Murray score) and APACHE II severity of illness score. Volume-controlled ventilation (VCV) was chosen as the ventilation mode for the connected ventilator (Drager Evita 4). The ventilator parameters were adjusted according to the driving pressure, with the optimal PEEP and tidal volume of the patients selected for ventilation. The VCV mode was maintained during the recruitment manoeuvre. At each 40 s increment, PEEP was then increased from 5 to 20 cmH_2_O by 5 cmH_2_O. The recruitment manoeuvre was followed by a decremental PEEP trial, which was initiated at a PEEP of 20 cmH_2_O. During this trial, with the same respiratory rate, PEEP was sequentially reduced from 20 to 5 cmH_2_O by 2 cmH_2_O every 30 s. Driving pressure was measured by subtracting the PEEP from the plateau pressure at the end of each step. Optimal PEEP was defined as a PEEP that produced the lowest driving pressure [17]. After randomization, esketamine with midazolam (esketamine group) or remifentanil with midazolam (control group) was selected according to the assigned groups. The two groups were administered medication via continuous intravenous infusion. The loading dose of esketamine was 0.5 mg/kg, followed by a maintenance dose of 0.125–0.15 mg/kg·h. In the case of midazolam, the loading dose was 0.3 mg/kg, while the maintenance dose was 0.02–0.1 mg/kg·h. Additionally, the loading dose of the remifentanil injection was 0.5 µg/kg, and the maintenance dose was 0.02–0.15 µg/kg·min. The RASS score was applied to the two groups, with a target sedation level of − 3 points. Similarly, the CPOT score was adopted in both groups, with the target analgesia level set as 3 points. Subsequent assessments were performed every 4 h to ensure sustained target sedation and analgesia levels. Continuation of sedation after the observation period was decided based on the disease progression and the clinician’s treatment strategy. At 2 h following enrollment, all patients underwent bronchoscopy for sputum suction and bronchoalveolar lavage for microbiological testing. Additionally, budesonide hydrochloride was administered by nebulized inhalation (times/12 h). The attending physician formulated the patient fluid resuscitation and antibiotic regimens based on the daily results of the cardiac ultrasound, fluid responsiveness experiments, and microbiological examinations.

### Outcomes

The primary outcome was C_st_ at 72 h after drug administration. Other respiratory mechanics parameters including R_aw_, P_plat_, and P_peak_ also were measured. The secondary outcome variables were PaO_2_/FiO_2_ ratio, hemodynamic parameters, usage dose of vasopressor drugs, total ICU length of stay, and mechanical ventilation duration.

Before the measurement process, the ventilator parameters were set, and the patients were suctioned to ensure airway patency. After 30 min, two researchers assessed the respiratory mechanics using the airway obstruction method [18]. In this method, a single dose of rocuronium bromide at 0.3 mg/kg was administered to inhibit the spontaneous breathing of the patients, facilitating accurate estimations of the respiratory mechanics parameters. Neuromuscular blocking agents were utilized only during the measurements. During the measurement process in the VCV mode, the pressure time waveform was displayed on the ventilator function menu. Subsequently, the inhalation hold button was pressed for 3–5 s to evenly distribute the gas in the lungs. The ventilator interface was then frozen to measure the P_peak_ and P_plat_, and the pressure difference between P_peak_ and P_plat_ to overcome R_aw_ was used to calculate R_aw_, wherein R_aw_ = (P_peak_ − P_plat_)/flow rate. During the breath-hold, no gas flow or resistance was generated in the airway. Thus, P_plat_ was completely used to overcome the elastic resistance of the lungs and chest wall, i.e., Cst = tidal volume/(P_plat_ − PEEP). The measurements were conducted before medication administration and at 24, 48, and 72 h post-medication, with the best C_st_ value at each time point being recorded. The averages of the values obtained by the two researchers were used as the final results. Furthermore, the PaO_2_/FiO_2_ ratio was calculated by performing a blood gas analysis of arterial blood samples (1 mL) drawn from the arterial catheter at each time point. Mean arterial pressure and heart rate were also recorded at each time point.

### Statistical analysis

The required sample size for this study was estimated based on the results of previously published clinical studies [19]. During the pilot phase of this study, the mean C_st_ of patients with ARDS admitted to our center was 40 mL/cmH_2_O. Esketamine administration led to a 20% increase in C_st_ among patients with ARDS, with α = 0.05 and power = 0.9. According to the PASS software calculation, 50 patients (25 in each group) were required to be included.

Statistical analyses were performed using SPSS 25.0. The normality of the measurement data was analyzed utilizing the Shapiro–Wilk test. Measurement data with normal distribution were expressed as mean ± standard deviation ($\overline{{x}}\pm{{s}}$), with the two independent samples t-test used for between-group comparisons and repeated-measures ANOVA for assessing repeated measurement data. Non-normally distributed measurement data were presented as median and interquartile range and examined by applying the Mann–Whitney *U* test. Count data were described as rates and examined utilizing the chi-square test or Fisher’s exact probability method. The multiple-sample rank sum test was employed to compare rank information. All analyses were two-sided tests at an α-level of 0.05, with a *P*-value of < 0.05 considered statistically significant.

## Results

**Fig. 1:**
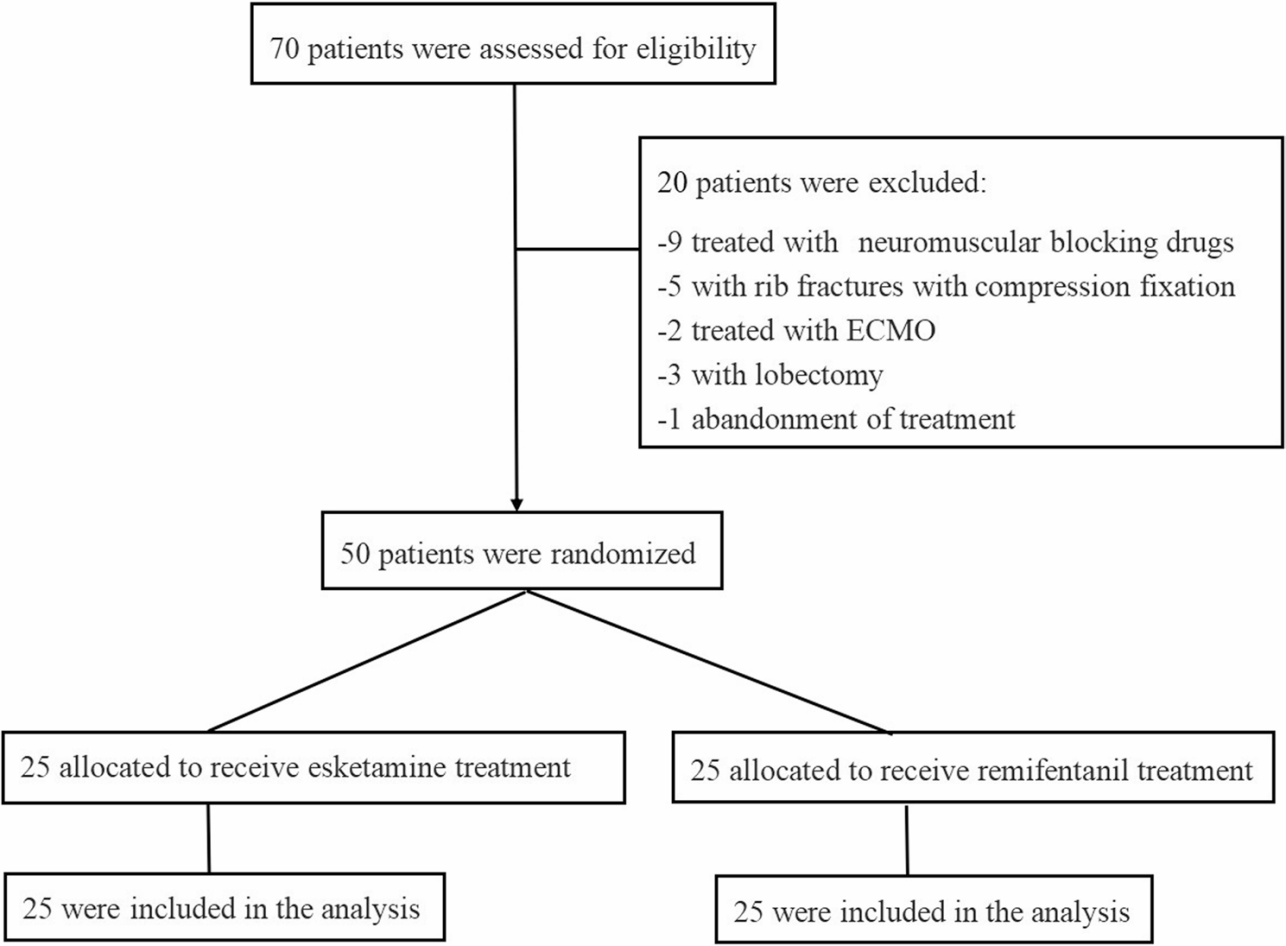
Study flowchart

This study screened 70 patients with ARDS who were admitted to the ICU of Tangdu Hospital of Air Force Medical University from April 2023 to December 2023. Ultimately, 50 patients were enrolled in this randomized controlled study (Fig. 1).

Baseline data of the patients in the esketamine and control groups are presented in Table 1. The lung injury score (Murray score) and APACHE II severity of illness score did not differ between the two groups. Respiratory parameters (including tidal volume, respiratory rate, PEEP, P_plat_, C_st_, and R_aw_ values) were similar between the two groups at baseline. The PaO_2_/FiO_2_ ratio, which indicated ARDS severity, did not-differ between the esketamine and control groups (192.4 ± 69.7 vs. 208.9 ± 65.0, *P* = 0.389).

**Table 1:** Baseline characteristics of patients

Characteristic	Esketamine (*N* = 25)	Control (*N* = 25)	*P*
Age, yrs	59.8 ± 17.7	60.1 ± 12.6	0.943
Gender, Male, n	15	19	0.234
APACHEII	21.0 ± 4.8	23.8 ± 6.2	0.089
Murray	7.8 ± 2.4	7.8 ± 2.0	1.000
Balance of in-out volu, ml	-168.2	56.5	0.664
Respiratory parameters			
Expired VT, ml	404.0 ± 34.2	414.0 ± 24.7	0.223
PEEP, cmH_2_O	7.5 ± 1.5	7.5 ± 1.1	1.000
Pplat, cmH_2_O	17.2 ± 3.0	18.7 ± 4.4	0.156
PIP, cmH_2_O	28.5 ± 5.2	30.8 ± 5.7	0.140
Pulmonary compliance, ml/cmH_2_O	41.6 ± 11.0	40.6 ± 11.0	0.744
Airway resistance, cm H_2_O·L^−1^·s^−1^	11.4 ± 3.3	11.4 ± 2.9	0.916
Respiratory rate, min^−1^	18.5 ± 1.6	18.8 ± 1.4	0.509
PaO_2_/FiO_2_ ratio	192.4 ± 69.7	208.9 ± 65.0	0.389
PH	7.41 ± 0.07	7.38 ± 0.17	0.261
Hemodynamic parameters			
Mean arterial pressure, mmHg	85.0 ± 9.1	84.6 ± 12.8	0.898
Heart rate, beats·min^−1^	83.0 ± 12.6	86.9 ± 15.6	0.339
Lactic acid, mmol·L^−1^	2.2 ± 2.0	2.4 ± 3.4	0.764
Medical history, N (%)			
Hypertension	5 (20%)	8 (32%)	0.344
Diabetes	5 (20%)	6 (24%)	0.739
Dyslipidemia	2 (8%)	1 (4%)	0.561
Chronic respiratory disease	2 (8%)	2 (8%)	1.000
Chronic renal failure	3 (12%)	1 (4%)	0.307
Alcohol dependence	4 (16%)	3 (12%)	0.691
Active tobacco smoking	7 (28%)	6 (24%)	0.753
Cause of ARDS, N (%)			
Pneumonia	12 (48%)	12 (48%)	1.000
Extrapulmonary causes	13 (52%)	13 (52%)	1.000

Data are expressed as mean ± standard deviation

*Abbreviations*: *PBW* predicted body weight, *APACHE II* Acute Physiology and Chronic Health Score, *Murray* lung injury score

### Effects of esketamine on respiratory mechanics

The normality and chi-square tests for C_st_ and R_aw_ results at different time points in the two groups demonstrated that these results obeyed normal and chi-square distributions (*P* > 0.05). However, Mauchly’s spherical hypothesis test revealed that the variance-covariance matrices of C_st_ and R_aw_ were different (χ^2^ = 18.992, *P* = 0.002; χ^2^ = 29.709, *P* = 0.001, respectively), and the results were corrected by applying the Greenhouse–Geisser method.

**Fig. 2:**
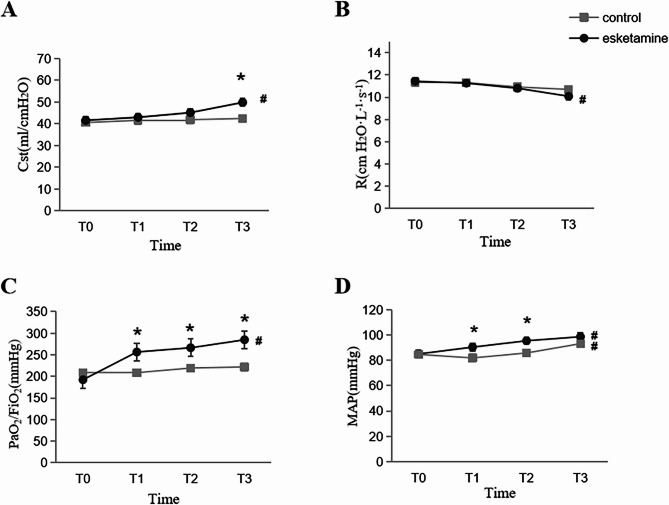
**A** pulmonary compliance levels at different time points in patients of two groups; **B** Airway resistance levels at different time points in patients of two groups; **C** PaO_2_/FiO_2_ ratio levels at different time points in patients of two groups; **D** Mean arterial pressure levels at different time points in patients of two groups. Time T0 represents the time of pre-dose, T1 represents the time of 24 h post-dose, T2 represents the time of 48 h post-dose, T3 represents the time of 72 h post-dose. * indicates comparison between groups at a certain time point, *P* < 0.05. # indicates comparison within groups over time, *P* < 0.05. Measurement data with normal distribution were expressed as mean ± standard deviation, repeated-measures ANOVA for assessing repeated measurement data

After adjustment, the repeated-measures ANOVA results indicated significant time (*P* < 0.001) and interaction effects (*P* = 0.001) but no such significance for between-group effect (*P* = 0.303) for C_st_ in the esketamine and control groups (Table 2), implying that the effect of time on C_st_ varied with different sedation and analgesia regimens. Post hoc pairwise comparisons with Bonferroni correction showed no significant differences in C_st_ between the two groups at 24 and 48 h post-treatment; however, C_st_ at 72 h post-treatment was significantly higher in the esketamine group than in the control group (49.8 ± 13.7 vs. 42.4 ± 11.9, *P* = 0.049) (Table 3), the 95% confidence interval for the esketamine group mean is (44.1, 55.5) and the 95% confidence interval for the control group mean is (37.5, 47.4). Additionally, prolonged analgesic sedation resulted in an increasing tendency for C_st_ in the two groups (Fig. 2A). This increasing tendency in the C_st_ had a different range between the two groups, with a more pronounced increase in the esketamine group. Moreover, the elevation in C_st_ remained significantly higher in the esketamine group compared with the control group from baseline to 72 h post-treatment (8.2 ± 6.8 mL/cmH_2_O vs. 1.9 ± 9.3 mL/cmH_2_O; *P* = 0.005). Furthermore, a significant time effect was observed for R_aw_ in the two groups (*P* = 0.009), whereas no such significance was detected for between-group (*P* = 0.788) or interaction effects (*P* = 0.704) (Table 2). In the case of R_aw_, a decreasing trend was demonstrated in the two groups (Fig. 2B), with a relatively greater decrease in the esketamine group. This reduction in R_aw_ was significantly higher in the esketamine group than in the control group from baseline to 72 h post-treatment (1.3 ± 3.0 vs. 0.6 ± 2.5; *P* = 0.030). Finally, the plateau pressure and peak airway pressure values in the esketamine group were lower than those in the control group (plateau pressur: 15.6 ± 3.1 vs. 17.7 ± 4.2; *P* = 0.047; peak airway pressure: 25.7 ± 3.8 vs. 28.5 ± 4.7; *P* = 0.024) at 72 h post-treatment (Table 3).

**Table 2:** Repeated measures ANOVA of pulmonary compliance, airway resistance, and oxygenation index in two groups of patients

Variable	Time effect		Between-group effect		Interactive effect	
	F	*P*	F	*P*	F	*P*
C_st_	12.546	< 0.001^*^	1.083	0.303	5.642	0.003^*^
R_aw_	4.018	0.022^*^	0.073	0.788	0.469	0.637
PaO_2_/FiO_2_	10.757	< 0.001^*^	4.191	0.046^*^	6.438	0.001^*^

Repeated-measures ANOVA was analyzed using the Greenhouse-Gessler correction because the assumption of sphericity was not met

*P < 0.05 difference is statistically significant

**Table 3:** Comparison of C_st_, R_aw_, P_plat_, P_peak_, PaO_2_/FiO_2_ ratio, MAP and HR levels between the two groups (mean ± standard deviation)

Variable	Group	T0	T1	T2	T3	*P*
C_st_	esketamine	41.6 ± 11.0	43.0 ± 12.8	45.1 ± 14.0	49.8 ± 13.8	< 0.001^*^
	control	40.6 ± 11.0	41.6 ± 9.2	41.9 ± 8.6	42.4 ± 11.9	0.689
	*P*	0.744	0.660	0.327	0.049^*^	
R_aw_	esketamine	11.4 ± 3.3	11.3 ± 2.7	10.8 ± 2.7	10.1 ± 2.0	0.022^*^
	control	11.4 ± 2.9	11.3 ± 2.3	10.9 ± 2.5	10.7 ± 2.5	0.534
	*P*	0.916	0.948	0.875	0.334	
P_plat_	esketamine	17.2 ± 3.0	17.8 ± 3.6	16.7 ± 4.3	15.6 ± 3.1	0.013^*^
	control	18.7 ± 4.3	18.7 ± 3.7	18.3 ± 3.6	17.7 ± 4.2	0.444
	*P*	0.156	0.368	0.157	0.047^*^	
P_peak_	esketamine	28.5 ± 5.2	29.0 ± 4.5	27.3 ± 4.6	25.7 ± 3.8	0.001^*^
	control	30.8 ± 5.7	30.0 ± 4.5	29.2 ± 4.4	28.5 ± 4.7	0.163
	*P*	0.140	0.471	0.138	0.024^*^	
PaO_2_/FiO_2_	esketamine	192.4 ± 69.7	256.6 ± 89.1	266.4 ± 88.5	284.9 ± 84.5	< 0.001^*^
	control	208.9 ± 65.0	208.7 ± 61.5	219.5 ± 61.3	222.0 ± 68.5	0.661
	*P*	0.389	0.032^*^	0.034^*^	0.006^*^	
MAP	esketamine	85.0 ± 9.1	90.4 ± 8.8	95.4 ± 10.0	98.6 ± 10.0	< 0.001^*^
	control	84.6 ± 12.8	81.9 ± 10.7	85.8 ± 11.7	93.1 ± 11.7	0.004^*^
	*P*	0.898	0.003^*^	0.003^*^	0.083	
HR	esketamine	83.0 ± 12.6	89.2 ± 10.0	92.3 ± 10.9	94.2 ± 10.2	0.011^*^
	control	86.9 ± 15.6	84.9 ± 13.4	85.6 ± 15.4	87.4 ± 13.7	0.674
	*P*	0.339	0.199	0.080	0.052	

Esketamine (*N* = 25), control (*N* = 25). Time T0 represents the time of pre-dose, T1 represents the time of 24 h post-dose, T2 represents the time of 48 h post-dose, T3 represents the time of 72 h post-dose

Measurement data with normal distribution were expressed as mean ± standard deviation, repeated-measures ANOVA for assessing repeated measurement data

^*^P < 0.05 difference is statistically significant, pairwise comparisons are Bonferroni-corrected

### Effect of esketamine on PaO_2_/FiO_2_ ratio

The PaO_2_/FiO_2_ ratio was significantly higher in the esketamine group than in the control group. Repeated-measures ANOVA analyses revealed significant between-group effects (*P* = 0.046), time effects (*P* < 0.001), and interaction effects (*P* < 0.001) for the PaO_2_/FiO_2_ ratio in two groups (Table 2), suggesting that the effect of time on the PaO_2_/FiO_2_ ratio varied with sedation and analgesia regimens. The post hoc pairwise comparison results showed that the PaO_2_/FiO_2_ ratio of the esketamine group were significantly higher than those of the remifentanil group at 24 h (256.7 ± 89.1 vs. 208.7 ± 61.5, *P* = 0.032), 48 h (266.4 ± 88.5 vs. 219.5 ± 61.3, *P* = 0.034), and 72 h (284.9 ± 84.5 vs. 222.0 ± 68.5, *P* = 0.006) post-medication administration. Lastly, the heightened PaO_2_/FiO_2_ ratio of the esketamine group remained significantly higher than that of the remifentanil group from baseline to 72 h post-treatment (92.6 ± 81.7 vs. 13.1 ± 89.2; *P* = 0.002) (Fig. 2C).

### Hemodynamic effects of esketamine

The average arterial pressure and heart rate were higher while the cumulative dose of vasoactive drugs was lower in the esketamine group than those in the remifentanil group. Mean arterial pressure tended to increase over time in the two groups (Fig. 2D), with this increase exhibiting statistical significance in the esketamine group (*P* < 0.001). Moreover, the mean arterial pressure was found to be significantly higher in the esketamine group than in the remifentanil group at 24 and 48 h post-treatment (90.4 ± 8.8 vs. 81.9 ± 10.7 *P* = 0.003 and 95.4 ± 10.0 vs. 85.8 ± 11.7, *P* = 0.003, respectively) (Table 3). Although heart rate was higher in the esketamine group than in the remifentanil group, this difference was not statistically significant. Finally, the cumulative dose of vasoactive drugs was significantly lower in the esketamine group than in the remifentanil group (*P* = 0.05) (Table 4).

**Table 4:** Comparison of clinical outcomes between the two groups ((mean ± standard deviation)

Group	*N*	Accumulation of vasoactive drugs(mg)	Duration of mechanical ventilation(h)	Duration of ICU stay(d)
Esketamine	25	33.1 ± 51.9	163.2 ± 85.7	12.6 ± 8.4
Control	25	69.8 ± 75.7	195.0 ± 80.6	13.2 ± 5.8
t		-2.000	-1.280	-0.254
*P*		0.050	0.207	0.800

### Comparison of clinical efficacy

There was no significant between-group difference in the duration of mechanical ventilation (163.2 ± 85.7 h (esketamine) vs. 195.0 ± 80.6 h (control group), *P* = 0.207). Conversely, the ICU length of stay did not differ between the esketamine group and control group (12.6 ± 8.4 days vs. 13.2 ± 5.8 days, *P* = 0.800) (Table 4).

## Discussion

This study compared esketamine/midazolam and remifentanil/midazolam in mechanically ventilated patients with ARDS. The results showed that while R_aw_ did not significantly differ between groups at any time point, the esketamine group exhibited numerically lower values. Notably, the esketamine group demonstrated statistically significant improvement in C_st_ at 72 h post-dose (42.4 vs. 49.8, *P* < 0.05), though the clinical significance of this modest improvement warrants further investigation. Additionally, the esketamine group showed consistently higher PaO_2_/FiO_2_ ratios than control group at 24 h, 48 h, and 72 h post-dose (*P* < 0.05). These findings suggest that esketamine may have potential benefits for respiratory mechanics and oxygenation in ARDS patients, but the observed effects on C_st_ should be interpreted cautiously given the relatively small magnitude of difference.

The pathophysiological hallmarks of ARDS encompass diminished lung volume and reduced C_st_ [20]. Additionally, animal models of ARDS indicate that R_aw_ is significantly elevated during diffuse alveolar injury due to factors such as increased airway secretion, diminished lung volume, vagus reflex, and airway hypersensitivity [21]. Currently, no specific treatment method is available for ARDS, with invasive mechanical ventilation being the primary intervention. On the basis of treating the underlying disease, it is crucial to minimize the iatrogenic damage from medications and mechanical ventilation during the treatment period. Furthermore, the long-term use of opioid drugs is well-documented to exert adverse on the respiratory, immune, and digestive systems [22, 23]. Against this backdrop, the present study was designed to investigate the impact of analgesia and sedation management during mechanical ventilation in patients with ARDS. Specifically, we compared the effects of an opioid analgesic (remifentanil) and a non-opioid analgesic (esketamine) on respiratory mechanics in mechanically ventilated patients with ARDS.

This study’s sedation protocol, employing continuous intravenous infusion of midazolam, was formulated based on multidimensional evidence-based considerations. Although current ARDS management guidelines recommend non-benzodiazepine agents as the preferred choice for light sedation strategies, the decision to use continuous midazolam infusion instead of propofol was ultimately made in consideration of this patient cohort’s unique hemodynamic characteristics and sedation requirements. From a pharmacokinetic perspective, midazolam possesses distinctive physicochemical properties. Its pKa value enables pH-dependent ion trapping under physiological conditions, forming highly lipophilic molecules that facilitate rapid blood-brain barrier penetration and central nervous system effects [24]. Under continuous infusion, the drug exhibits linear pharmacokinetics, with steady-state plasma concentrations directly proportional to infusion rates [25]. This characteristic allows for precise sedation titration guided by RASS scores. Regarding safety, this protocol incorporates a modified dosing strategy: co-administration of low-dose esketamine (0.125–0.15 mg/kg/h) to enhance analgesia and reduce total midazolam requirements. This approach is derived from Lundy et al.‘s “balanced sedation” theory, which involves synergistic modulation of NMDA receptors and the GABAergic system [26]. This study employs a closed-loop feedback control system, dynamically adjusting infusion rates based on real-time RASS assessments to minimize drug accumulation risks.

Our study used esketamine/midazolam in mechanically ventilated ARDS patients, showing that compared with the control group, esketamine group exhibited a significant increase in C_st_, P_plat_ and P_peak_ at 72 h post-dose. These finding may be attributed to the pharmacological effects of esketamine which include inhibition of norepinephrine reuptake, excitation of the sympathetic nervous system, and relaxation of bronchial smooth muscle. This finding is consistent with those reported in previous clinical studies. Elamin et al. found that ketamine administration for pain relief in ICU patients could increase C_st_ and reduce R_aw_ [19]. Another study by Aiman Suleiman et al. demonstrated that ketamine could enhance inspiratory flow and diminish inspiratory respiratory work in mechanically ventilated patients in the ICU following surgical procedure [15]. Children with refractory bronchospasm undergoing mechanical ventilation have also shown a favorable response to continuous ketamine infusion, achieving significant improvements in C_st_ and oxygenation [14]. Similarly, Farhad Heshmati et al. showed that ketamine use could diminish airway peak pressure and suggested that it may also improve respiratory mechanics parameters [27].

At 24 h and 48 h post-dose, there were no significant differences in C_st_, P_plat_ and P_peak_ between the two groups. However, the C_st_ values were higher, and the P_plat_ and P_peak_ were lower in the esketamine group. We speculate that this may be because the inflammatory response was particularly active within the first 48 h of ARDS onset, and the severe pathological state of lung injury masked the potential beneficial effects of esketamine on C_st_. Although esketamine has certain advantages, it cannot significantly improve C_st_ in a short period of time and has not yet demonstrated a clear superiority over remifentanil.

Moreover, our study results revealed a decreasing trend in R_aw_ in the esketamine group, with no statistical significance in these observations. Prior case reports have demonstrated that patients with severe refractory asthma treated with mechanical ventilation may benefit from ketamine therapy, exhibiting alleviated bronchospasm and enhanced oxygenation [28]. Conversely, several studies have reported contrasting findings. For example, a randomized controlled study in 2020 showed that ketamine administration did not lead to a reduction in R_aw_ compared with fentanyl treatment at 3 h (0 ± 6 cmH_2_O/L·s, 3 ± 7.7 cmH_2_O/L·s; *P* = 0.16) and 24 h (− 3 ± 17 cmH_2_O/L·s, − 3.5 ± 13.7 cmH_2_O/L·s; *P* = 0.73) after treatment [7]. Although a decreasing trend in R_aw_ after esketamine administration was observed in this study, this reduction was not statistically significant. The factors that could explain the non-significant results include the increased salivary gland and airway secretions caused by esketamine and the failure to timely aspirate oropharyngeal secretions in clinical care. Furthermore, in this study, we were unable to effectively control for confounding factors affecting R_aw_, such as interpatients airway variability, the diverse suctioning techniques utilized by the nursing staff, and the heterogeneous patient responses to the suctioning stimulus. Additionally, the small sample size of this study may have served as another potential confounding factor.

ARDS is usually associated with hemodynamic instability, a prominent mortality-associated factor [29]. Adequate hemodynamics can maintain an appropriate ventilation-to-blood flow ratio and augment oxygenation. However, the underlying pathology of ARDS and mechanical ventilation factors pose a challenge to hemodynamics maintenance. Esketamine can reduce cardiac parasympathetic activity by blocking parasympathetic Na^+^ channels in the brainstem and hampering catecholamine reuptake, thereby elevating the heart rate and blood pressure. In this study, the esketamine group exhibited stabilized hemodynamics, heightened mean arterial pressure, and reduced use of vasoactive drugs compared with those of the control group. These findings are consistent with the results of a prospective study conducted by Reese et al., which revealed that esketamine treatment effectively reduced the dosage of vasopressor medications in mechanically ventilated patients with septic shock [30].

Esketamine reduces oxygen consumption, reduces man-machine antagonism through analgesia; improves C_st_ by dilating bronchial smooth muscle; and stabilizes hemodynamics by increasing blood pressure and heart rate. Therefore, esketamine optimizes ventilation-perfusion matching and enhances gas exchange, further improving oxygenation. This study also shown the PaO_2_/FiO_2_ ratio significant higher in the esketamine group compared with the control group (*P* < 0.05) at 24 h, 48 h, and 72 h post-dose. Improved oxygenation also facilitates weaning and extubation. However, no significant difference was observed in the duration of mechanical ventilation between the two groups, which may be attributed to the small sample size and the inconsistent progression of the underlying diseases.

This study has a few limitations that should be considered. First, this study was a single-center randomized controlled trial with a small sample size; consequently, some indicators only demonstrated trends rather than yielding statistically significant results. Further multicenter randomized controlled trials are required to confirm our findings. Second, although the sedation regimen used in this study deviates from current ARDS management guidelines, its adoption followed meticulous deliberation during the protocol design phase, as thoroughly justified in preceding sections. Third, an important methodological consideration is that our measurement of static respiratory system compliance inherently incorporates both lung and chest wall mechanics. While we observed significant changes in C_st_, the absence of esophageal pressure measurements precluded differentiation between pulmonary and extrapulmonary contributions. Factors such as abdominal distension, chest wall edema, or neuromuscular blockade effects might have influenced chest wall compliance, thereby affecting C_st_ measurements independent of lung tissue changes. Therefore, transpulmonary pressure, airway closure pressure, lung driving pressure, time constants, and other indicators should be incorporated in future research.

In conclusion, our randomized controlled trial revealed that compared with remifentanil/midazolam, esketamine/midazolam in mechanically ventilated patients with ARDS decreased P_peak_ and P_plat_, increased C_st_ and improved PaO_2_/FiO_2_ ratio 72 h after administration. All these new findings suggest that esketamine combined with midazolam may be a more favorable sedation and analgesia regimen than remifentanil combined with midazolam for critically ill patients in the ICU. Nevertheless, future multicenter clinical studies with large samples are warranted to establish the applicability of esketamine treatment during mechanical ventilation in patients with ARDS.

## Supplementary Information

Supplementary Material 1.

## Data Availability

The datasets used and/or analyzed during the currentstudy are available from the corresponding author uponreasonable request.

## References

[1] Bellani G, Laffey JG, Pham T, Fan E, Brochard L, Esteban A, Gattinoni L, van Haren F, Larsson A, McAuley DF, et al. Epidemiology, patterns of care, and mortality for patients with acute respiratory distress syndrome in intensive care units in 50 countries. JAMA. 2016;315(8):788–800. 26903337 10.1001/jama.2016.0291

[2] Bates JHT, Smith BJ. Ventilator-induced lung injury and lung mechanics. Ann Transl Med. 2018;6(19):378. 10.21037/atm.2018.06.29 PMC6212358 30460252

[3] Mauri T, Lazzeri M, Bellani G, Zanella A, Grasselli G. Respiratory mechanics to understand ARDS and guide mechanical ventilation. Physiol Meas. 2017;38(12):R280–h303. 28967868 10.1088/1361-6579/aa9052

[4] Chanques G, Constantin JM, Devlin JW, Ely EW, Fraser GL, Gélinas C, Girard TD, Guérin C, Jabaudon M, Jaber S, et al. Analgesia and sedation in patients with ARDS. Intensive Care Med. 2020;46(12):2342–56. 33170331 10.1007/s00134-020-06307-9 PMC7653978

[5] Gitti N, Renzi S, Marchesi M, Bertoni M, Lobo FA, Rasulo FA, Goffi A, Pozzi M, Piva S. Seeking the light in intensive care unit sedation: the optimal sedation strategy for critically ill patients. Front Med. 2022;9:901343. 10.3389/fmed.2022.901343 PMC9265444 35814788

[6] Kiyatkin EA. Respiratory depression and brain hypoxia induced by opioid drugs: morphine, oxycodone, heroin, and fentanyl. Neuropharmacology. 2019;151:219–26. 30735692 10.1016/j.neuropharm.2019.02.008 PMC6500744

[7] Chen R, Tang LH, Sun T, Zeng Z, Zhang YY, Ding K, Meng QT. Mechanism and management of fentanyl-induced cough. Front Pharmacol. 2020;11:584177. 33324214 10.3389/fphar.2020.584177 PMC7723435

[8] Wiese AD, Griffin MR, Schaffner W, Stein CM, Greevy RA, Mitchel EF, Grijalva CG. Long-acting opioid use and the risk of serious infections: a retrospective cohort study. Clin Infect Dis. 2019;68(11):1862–9. 10.1093/cid/ciy809 PMC6522680 30239630

[9] Yan Y, Chen Y, Zhang X. The effect of opioids on gastrointestinal function in the ICU. Crit Care (London England). 2021;25(1):370. 10.1186/s13054-021-03793-1 PMC8543814 34689805

[10] Devlin JW, Skrobik Y, Gélinas C, Needham DM, Slooter AJC, Pandharipande PP, Watson PL, Weinhouse GL, Nunnally ME, Rochwerg B, et al. Clinical practice guidelines for the prevention and management of pain, agitation/sedation, delirium, immobility, and sleep disruption in adult patients in the ICU. Crit Care Med. 2018;46(9):e825–73. 30113379 10.1097/CCM.0000000000003299

[11] Schwenk ES, Viscusi ER, Buvanendran A, Hurley RW, Wasan AD, Narouze S, Bhatia A, Davis FN, Hooten WM, Cohen SP. Consensus guidelines on the use of intravenous ketamine infusions for acute pain management from the American society of regional anesthesia and pain medicine, the American academy of pain medicine, and the American society of anesthesiologists. Reg Anesth Pain Med. 2018;43(5):456–66. 29870457 10.1097/AAP.0000000000000806 PMC6023582

[12] Zhang XX, Zhang NX, Liu DX, Ding J, Zhang YN, Zhu ZQ. Research advances in the clinical application of esketamine. Ibrain. 2022;8(1):55–67. 37786420 10.1002/ibra.12019 PMC10528803

[13] Hirota K, Lambert DG. Ketamine; history and role in anesthetic pharmacology. Neuropharmacology. 2022;216:109171. 35764129 10.1016/j.neuropharm.2022.109171

[14] Youssef-Ahmed MZ, Silver P, Nimkoff L, Sagy M. Continuous infusion of ketamine in mechanically ventilated children with refractory bronchospasm. Intensive Care Med. 1996;22(9):972–6. 8905436 10.1007/BF02044126

[15] Suleiman A, Santer P, Munoz-Acuna R, Hammer M, Schaefer MS, Wachtendorf LJ, Rumyantsev S, Berra L, Chamadia S, Johnson-Akeju O, et al. Effects of ketamine infusion on breathing and encephalography in spontaneously breathing ICU patients. J Intensive Care Med. 2023;38(3):299–306. 35934953 10.1177/08850666221119716

[16] Ranieri VM, Rubenfeld GD, Thompson BT, Ferguson ND, Caldwell E, Fan E, Camporota L, Slutsky AS. Acute respiratory distress syndrome: the Berlin definition. JAMA. 2012;307(23):2526–33. 22797452 10.1001/jama.2012.5669

[17] Kim YJ, Kim BR, Kim HW, Jung JY, Cho HY, Seo JH, Kim WH, Kim HS, Hwangbo S, Yoon HK. Effect of driving pressure-guided positive end-expiratory pressure on postoperative pulmonary complications in patients undergoing laparoscopic or robotic surgery: a randomised controlled trial. Br J Anaesth. 2023;131(5):955–65. 37679285 10.1016/j.bja.2023.08.007

[18] Grasselli G, Brioni M, Zanella A. Monitoring respiratory mechanics during assisted ventilation. Curr Opin Crit Care. 2020;26(1):11–7. 31738232 10.1097/MCC.0000000000000681

[19] Elamin E. Impact of ketamine on dynamic compliance and airway resistance of sedated and mechanically ventilated ICU patients. Cirit Care. 2009;13(Suppl 1):P404.

[20] Meyer NJ, Gattinoni L, Calfee CS. Acute respiratory distress syndrome. Lancet (London England). 2021;398(10300):622–37. 34217425 10.1016/S0140-6736(21)00439-6 PMC8248927

[21] Christley S, Emr B, Ghosh A, Satalin J, Gatto L, Vodovotz Y, Nieman GF, An G. Bayesian inference of the lung alveolar spatial model for the identification of alveolar mechanics associated with acute respiratory distress syndrome. Phys Biol. 2013;10(3):036008. 23598859 10.1088/1478-3975/10/3/036008

[22] Carron M, Tamburini E, Linassi F, Pettenuzzo T, Boscolo A, Navalesi P. Non-opioid analgesics and adjuvants after surgery in adults with obesity: systematic review with network meta-analysis of randomized controlled trials. J Clin Med. 2024;13(7):1–18. 10.3390/jcm13072100 PMC11012569 38610865

[23] Carron M, Tamburini E, Linassi F, Pettenuzzo T, Boscolo A, Navalesi P. Efficacy of nonopioid analgesics and adjuvants in multimodal analgesia for reducing postoperative opioid consumption and complications in obesity: a systematic review and network meta-analysis. Br J Anaesth. 2024;133(6):1234–49. 39366846 10.1016/j.bja.2024.08.009

[24] Reves JG, Fragen RJ, Vinik HR, Greenblatt DJ. Midazolam: pharmacology and uses. Anesthesiology. 1985;62(3):310–24. 3156545

[25] Fragen RJ. Pharmacokinetics and pharmacodynamics of Midazolam given via continuous intravenous infusion in intensive care units. Clin Ther. 1997;19(3):405–19. 9220206 10.1016/s0149-2918(97)80126-9

[26] Lundy JS, Adams R. Intravenous anesthesia. Am J Surg. 1936;34(3):559–70.

[27] Heshmati F, Zeinali MB, Noroozinia H, Abbacivash R, Mahoori A. Use of ketamine in severe status asthmaticus in intensive care unit. Iran J Allergy Asthma Immunol. 2003;2(4):175–80. 17301376

[28] Lau TT, Zed PJ. Does ketamine have a role in managing severe exacerbation of asthma in adults? Pharmacotherapy. 2001;21(9):1100–6. 11560199 10.1592/phco.21.13.1100.34618

[29] Vieillard-Baron A, Matthay M, Teboul JL, Bein T, Schultz M, Magder S, Marini JJ. Experts’ opinion on management of hemodynamics in ARDS patients: focus on the effects of mechanical ventilation. Intensive Care Med. 2016;42(5):739–49. 27038480 10.1007/s00134-016-4326-3

[30] Vankawala J, Naples G, Avila-Quintero VJ, Ramírez KL, Flores JM, Bloch MH, Dwyer JB. Meta-analysis: hemodynamic responses to Sub-anesthetic doses of ketamine in patients with psychiatric disorders. Front Psychiatry. 2021;12:549080. 33841195 10.3389/fpsyt.2021.549080 PMC8024485

